# Assessing the ecological niche and invasion potential of the Asian giant hornet

**DOI:** 10.1073/pnas.2011441117

**Published:** 2020-09-22

**Authors:** Gengping Zhu, Javier Gutierrez Illan, Chris Looney, David W. Crowder

**Affiliations:** ^a^Tianjin Key Laboratory of Animal and Plant Resistance, Tianjin Normal University, 300387 Tianjin, China;; ^b^Tianjin Key Laboratory of Conservation and Utilization of Animal Diversity, Tianjin Normal University, 300387 Tianjin, China;; ^c^Department of Entomology, Washington State University, Pullman, WA 99164;; ^d^Washington State Department of Agriculture, Olympia, WA 98501

**Keywords:** biological invasions, niche modeling, dispersal, ensemble forecasts

## Abstract

The Asian giant hornet (*Vespa mandarinia*) was recently detected in western British Columbia, Canada and Washington State, United States. *V. mandarinia* are an invasion concern due to their ability to kill honey bees and affect humans. Here, we used habitat suitability models and dispersal simulations to assess potential invasive spread of *V. mandarinia*. We show *V. mandarinia* are most likely to establish in areas with warm to cool annual mean temperature, high precipitation, and high human activity. The realized niche of introduced populations is small compared to native populations, suggesting introduced populations could spread into habitats across a broader range of environmental conditions. Dispersal simulations also show that *V. mandarinia* could rapidly spread throughout western North America without containment. Given its potential negative impacts and capacity for spread, extensive monitoring and eradication efforts throughout western North America are warranted.

The Asian giant hornet (*Vespa mandarinia*) is native to Asia (Fig. 1*A*), where it is a predator of arthropods, including honey bees (1). Attacks by *V. mandarinia* on beehives involve pheromone marking to recruit hornets, and rapid killing of workers (2). Japanese honey bees (*Apis cerana*) can counter these attacks, but *Apis mellifera* (European honey bee) lacks effective defenses (2).

**Fig. 1.**
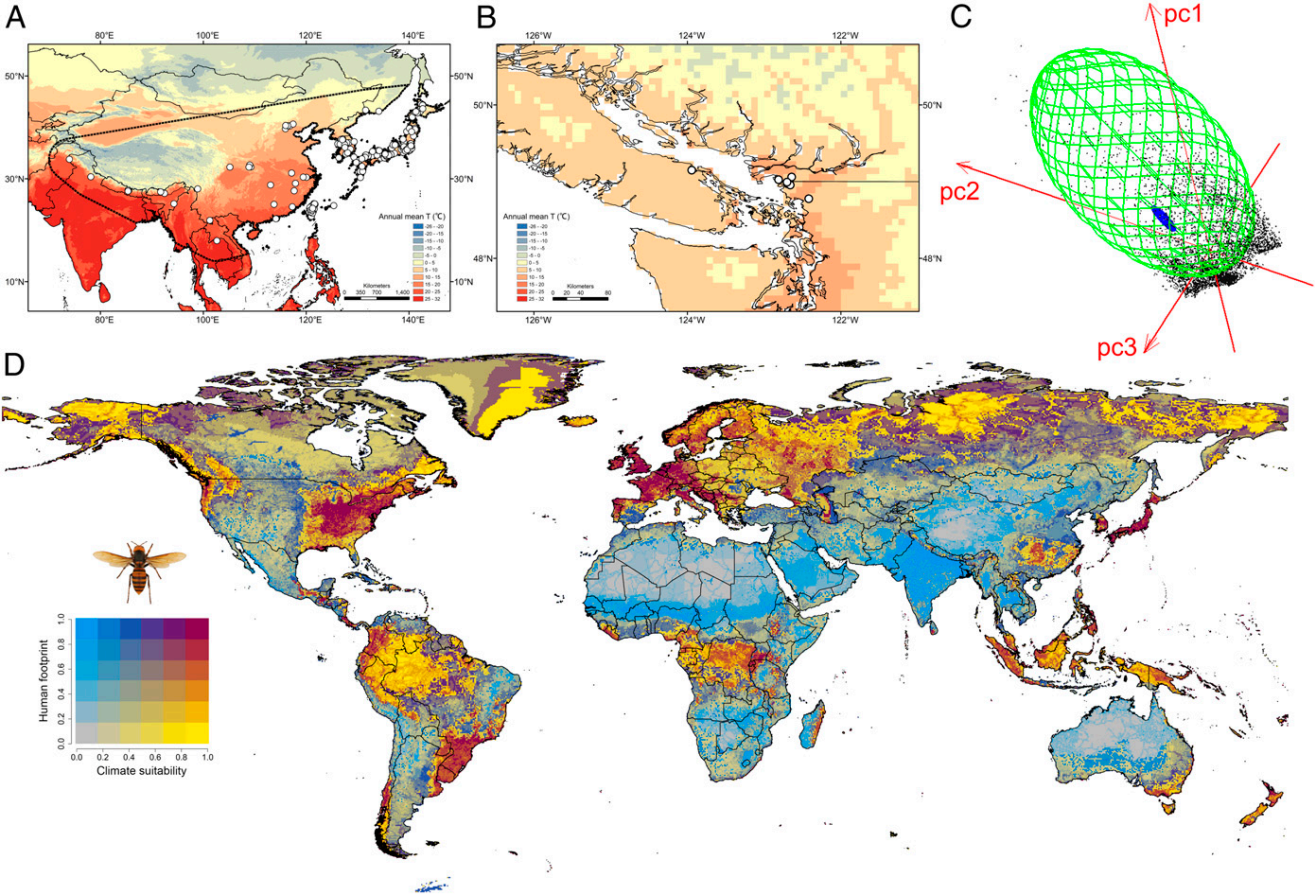
Present distribution of Asian giant hornet in (*A*) native and (*B*) introduced regions. In *A* points denote trimmed records used to fit models. (*C*) Realized niche occupied by native and introduced populations shown as minimum ellipsoid volumes. The green volume represents the native niche, the blue volume represents the introduced niche, and points denote environmental conditions globally. (*D*) Ensemble forecast of global habitat suitability. Increasing intensities of yellow represent increasing climate suitability, and increasing blue represent increasing severity of invasions due to human activity.

In September 2019, a *V. mandarinia* nest was found on Vancouver Island, British Columbia, Canada; later that year, four workers were found in Washington State, United States. In 2020, three additional queens were found (Fig. 1*B*) (3). Introduction of *V. mandarinia* in North America is concerning because honey bees are highly vulnerable to hornets (2). *V. mandarinia* is also medically important, delivering painful stings with cytolytic venom. It is currently unclear if *V. mandarinia* is established in North America and efforts are underway to identify introductions and prevent spread (3).

Mitigation efforts for *V. mandarinia* would be most effective if global habitat suitability, and potential dispersal into areas with high human activity, were better characterized (4). Here we modeled responses of *V. mandarinia* to climatic variables and simulated potential invasive spread. Our results can guide monitoring and eradication efforts for this invader.

## Results and Discussion

We first developed an ensemble model to examine habitat suitability for *V. mandarinia*. Our model had good discriminability (area under the curve = 0.95, true skill statistic = 0.34), and detections in North America have occurred in areas predicted to have highly suitable habitat (Fig. 1 *B* and *D*). Regions with low to warm temperatures and high precipitation appear most suitable for *V. mandarinia* (Fig. 1*D*). Such areas occur across western North America, eastern North America, Europe, northwestern and southeastern South America, central Africa, eastern Australia, and New Zealand. These regions all have high human activity, suggesting they could be strongly impacted by invasions (Fig. 1*D*); human activity could also facilitate invasions by *V. mandarinia* (5). However, most of central North America and California, which have considerable crop acreage that rely on *A. mellifera* pollination (6), are less suitable habitats. These predictions were based solely on abiotic factors; while biotic factors such as species interactions or evolution might affect invasions by *V. mandarinia* (7), such biotic data were not available.

**Fig. 2.**
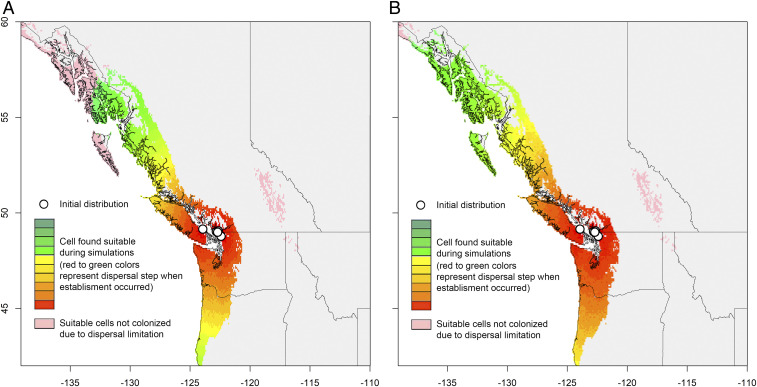
Estimated expansion of *V. mandarinia* over 20 y in western North America under two dispersal scenarios: (*A*) short-distance dispersal only and (*B*) both short- and long-dispersal distance dispersal. Each color represents two dispersal steps (total 20).

We next examined the realized niche of introduced and native *V. mandarinia* populations. Using minimum ellipsoid volumes, we show that the realized niche of introduced individuals in western North America is nested within the realized niche of native populations in Asia (Fig. 1*C*). This suggests the climatic niche of native populations was conserved during introductions, and that introduced populations could expand into regions across a broader range of conditions reflecting the native niche. Dispersal simulations also show high potential for spread (Fig. 2). When considering only short-distance dispersal, *V. mandarinia* could reach Oregon in 10 y and eastern Washington/British Columbia within 20 y (Fig. 2*A*). When accounting for long-distance human-mediated dispersal, expansion of *V. mandarinia* extended dramatically along coastal areas of British Columbia, with a faster rate of southern and eastern expansion (Fig. 2*B*). While these predictions are sensitive to the dispersal parameters, our results suggest that if *V. mandarinia* has a similar dispersal capacity to other invasive vespids; it could rapidly expand its invasive range throughout western North America absent coordinated mitigation efforts.

Ecological impacts of invasive vespids are hard to predict (7). Many transplanted Vespidae have minor impacts on native species, while others rapidly displace congeners (7, 8). Asian giant hornets prey on many insects (9) and could affect numerous species in North America. The broad habitat suitability and dispersal potential indicate negative ecological effects could ultimately be distributed over expansive areas. Spread of *V*. *mandarinia* could also affect beekeepers. Honey bees are used for pollination throughout North America, including areas predicted to have highly suitable habitat (6). Populations of *V. mandarinia* would likely prey on readily available hives, weakening or killing them. In Europe, *Vespa velutina* causes losses between 18% and 50% of beehives (10). Our results suggest western North America could face similar challenges if *V. mandarinia* spreads, forcing beekeepers to invest heavily in hornet management or relocate their operations.

In North America, monitoring programs have been developed to mitigate spread of *V. mandarinia*, incorporating tactics used in the hornet’s native range and experiences with invasive hornets in Europe and Asia. Our models identify regions with suitable *V. mandarinia* habitat, where monitoring can be focused to maximize efficiency. For example, Washington State’s citizen science program has more than 1,300 traps, with the vast majority in western Washington. Our models support this distribution, and indicate trapping in the arid central part of the state would be counterproductive. Our results also indicate that eradication efforts should assume a rapid expansion rate, such that trapping to detect introductions should be conducted throughout western North America. Given that *V. mandarinia* is not yet widely established, targeted monitoring and eradication efforts could prevent further spread of this damaging invader.

## Methods

### Environmental Factors Affecting *V. mandarinia*.

We assessed environmental factors mediating *V. mandarinia* occurrence using records gathered via the “spocc” package in R (10). We obtained 343 records from *V. mandarinia*’s native range; 119 were filtered out by enforcing a distance of 5 km between observations, resulting in 224 unique records (Fig. 1*A*). To determine climatic variables that constrain *V. mandarinia*, we considered temperature (annual mean, range, maximum of warmest and minimum of coldest months), precipitation (annual and wettest and driest months), and annual mean radiation. We relied on environmental variables because little is known about how other biological factors, such as species interactions or evolution, might impact *V. mandarinia*. For example, *V. mandarinia* predators are virtually unknown, and no congeners that might compete against *V. mandarinia* for resources occur in western North America (9).

### Realized Niche Modeling.

We assessed realized niches occupied by native and introduced *V. mandarinia* populations using minimum ellipsoid volumes, which depict niche breadth in three dimensions. We generated three environmental dimensions that summarized 90% of overall variation in the eight bioclimatic variables using principle component analysis in NicheA (http://nichea.sourceforge.net/).

### Ecological Niche Modeling.

We used an ensemble model to assess habitat suitability for *V. mandarinia* (11), which averaged predictions across five commonly used ecological niche models: 1) generalized additive, 2) generalized linear, 3) general boosted, 4) random forest, and 5) maximum entropy. Our approach included statistical models (1 and 2) that infer relationships between variables, and machine-learning models (3 to 5) that seek to obtain a general understanding of the data to make predictions. A common limitation of statistical models is that they require assumptions about the distribution of variables, whereas machine-learning models often suffer from overfitting and provide limited information about biological mechanisms affecting species distributions. However, while these differing approaches each have specific assumptions and limitations, by averaging predictions across models using a consensus method, ensemble models limit biases of any particular approach (11). Fifty percent of records were used for model training and 50% for validation. We used a “random” method in *biomod2* to select 10,000 pseudoabsences to improve model fit, from <400 m or >1,400 m elevations in “accessible” areas of *V. mandarinia* that were delimited by buffering minimum convex polygons of observed points at 400 km. We used area under the curve of receiver operating characteristic plots and true skill statistic to assess model fit.

Habitat modification has also been linked to invasiveness in some Vespidae, and areas with suitable climate and high human activity may be more susceptible to invasion (4, 7). We mapped human footprint, an indicator of human-mediated disturbances that combines population density and infrastructure (12) with climate suitability, using a bivariate approach (Fig. 1*D*).

### Dispersal Simulation.

*V. mandarinia* dispersal is mediated by queens. Dispersal behavior for most *Vespa* species are unknown, but invasive populations of the congener *V. velutina* have expanded by 78 km/y in France (13) and 18 km/y in Italy (14). To simulate *V. mandarinia* spread, we used the “MigClim” package in R (15). MigClim uses a dispersal time step, which we set as 1 y because queens form colonies once per year (1). We ran simulations for 20 y using habitat suitability from ensemble models and two dispersal scenarios: 1) short-distance only and 2) both short- and long-distance dispersal. Datasets to simulate dispersal had a resolution of 5 arcmin per pixel (∼5.5 km). We defined short-distance dispersal as less than ∼49.5 km (9 pixels), the average spread for *V. velutina* from two European studies (13, 14); short-distance dispersal was modeled with a dispersal kernel that assumed an exponential decline in the probability of movement at greater distances. We assumed that long-distance dispersal can occur up to ∼110 km (20 pixels), which reflects the maximum rate of spread observed for *V. velutina* in Europe (13). Within MigClim, long-distance dispersal events are generated with a defined probability and within a defined distance range. We set the dispersal probability as 1 to reflect that the assumption that 100% of source cells produce propagules in any given year; we also assumed long-distance dispersal occurred at distances greater than 9 pixels (49.5 km) but less than 20 pixels (110 km). While longer-distance dispersal via human activity is possible, we chose conservative values that likely capture the vast majority of human-mediated dispersal events (15). We assumed there are no geographic or environmental barriers to dispersal.

## Data Availability

The complete data file and methods are publicly available in Open Science Framework at https://osf.io/ed9az/.

## References

[1] Archer M. E., Taxonomy, distribution and nesting biology of the *Vespa mandarinia* group (HYM. Vespinae). Entomol. Mon. Mag. 131, 47–53 (1995).

[2] McClenaghan B.., Behavioral responses of honey bees, *Apis cerana* and *Apis mellifera*, to *Vespa mandarinia* marking and alarm pheromones. J. Apic. Res. 58, 141–148 (2019).

[3] United States Department of Agriculture, New Pest Response Guidelines for Asian Giant Hornet (Vespa Mandarinia), (United States Department of Agriculture, Animal and Plant Health Inspection Service, Plant Protection and Quarantine, 2019).

[4] Liebhold A. M., Tobin P. C., Population ecology of insect invasions and their management. Annu. Rev. Entomol. 53, 387–408 (2008). 17877456 10.1146/annurev.ento.52.110405.091401

[5] Hulme P. E., Trade, transport and trouble: Managing invasive species pathways in an era of globalization. J. Appl. Ecol. 46, 10–18 (2009).

[6] Calderone N. W., Insect pollinated crops, insect pollinators and US agriculture: Trend analysis of aggregate data for the period 1992-2009. PLoS One 7, e37235 (2012). 22629374 10.1371/journal.pone.0037235 PMC3358326

[7] Beggs J. R.., Ecological effects and management of invasive alien Vespidae. BioControl 56, 505–526 (2011).

[8] Crowder D. W., Snyder W. E., Eating their way to the top? Mechanisms underlying the success of invasive insect generalist predators. Biol. Invasions 12, 2857–2876 (2010).

[9] Matsuura M., Comparative biology of the five Japanese species of the genus *Vespa* (Hymenoptera, Vespidae). Bull. Faculty Agric. Mie Univ. 69, 1–131 (1984).

[10] R Core Team, A language for environment and statistical computing (Version R v4.0.0, R Foundation for Statistical Computing, Vienna, Austria, 2020).

[11] Thuiller W., Lafourcade B., Engler R., Araújo M. B., BIOMOD—A platform for ensemble forecasting of species distributions. Ecography 32, 369–373 (2009).

[12] Cabra-Rivas I., Saldaña A., Castro-Díez P., Gallien L., A multi-scale approach to identify invasion drivers and invaders’ future dynamics. Biol. Inv. 18, 411–426 (2016).

[13] Robinet C., Suppo C., Darrouzet E., Rapid spread of the invasive yellow-legged hornet in France: The role of human-mediated dispersal and the effects of control measures. J. Appl. Ecol. 54, 205–215 (2017).

[14] Bertolino S., Lioy S., Laurino D., Manino A., Porporato M., Spread of the invasive yellow-legged hornet *Vespa velutina* (Hymenoptera: Vespidae) in Italy. Appl. Entomol. Zool. 51, 589–597 (2016).

[15] Engler R., Hordijk W., Guisan A., The MIGCLIM R package—Seamless integration of dispersal constraints into projections of species distribution models. Ecography 35, 872–878 (2012).

